# Yeast artificial chromosomes employed for random assembly of biosynthetic pathways and production of diverse compounds in *Saccharomyces cerevisiae*

**DOI:** 10.1186/1475-2859-8-45

**Published:** 2009-08-13

**Authors:** Michael Naesby, Søren VS Nielsen, Curt AF Nielsen, Trine Green, Thomas Ø Tange, Ernesto Simón, Philipp Knechtle, Anders Hansson, Markus S Schwab, Olca Titiz, Christophe Folly, Roberto E Archila, Milena Maver, Stephan van Sint Fiet, Thiamo Boussemghoune, Michael Janes, A S Sathish Kumar, Shailendra P Sonkar, Partha P Mitra, V Ajai Kumar Benjamin, Nimitha Korrapati, Inala Suman, Esben H Hansen, Tanja Thybo, Neil Goldsmith, Alexandra Santana Sorensen

**Affiliations:** 1 Evolva SA, Hagmattstrasse 6, 4123 Allschwil, Switzerland; 2 Evolva Biotech Private Limited, 203-206, 2nd Floor, Tara Tycoon Building, Tarnaka, Secunderabad 500 017, India; 3 Evolva A/S, Bülowsvej 25, 1870 Frederiksberg C, Denmark

## Abstract

**Background:**

Natural products are an important source of drugs and other commercially interesting compounds, however their isolation and production is often difficult. Metabolic engineering, mainly in bacteria and yeast, has sought to circumvent some of the associated problems but also this approach is impeded by technical limitations. Here we describe a novel strategy for production of diverse natural products, comprising the expression of an unprecedented large number of biosynthetic genes in a heterologous host.

**Results:**

As an example, genes from different sources, representing enzymes of a seven step flavonoid pathway, were individually cloned into yeast expression cassettes, which were then randomly combined on Yeast Artificial Chromosomes and used, in a single transformation of yeast, to create a variety of flavonoid producing pathways. Randomly picked clones were analysed, and approximately half of them showed production of the flavanone naringenin, and a third of them produced the flavonol kaempferol in various amounts. This reflected the assembly of 5–7 step multi-species pathways converting the yeast metabolites phenylalanine and/or tyrosine into flavonoids, normally only produced by plants. Other flavonoids were also produced that were either direct intermediates or derivatives thereof. Feeding natural and unnatural, halogenated precursors to these recombinant clones demonstrated the potential to further diversify the type of molecules that can be produced with this technology.

**Conclusion:**

The technology has many potential uses but is particularly suited for generating high numbers of structurally diverse compounds, some of which may not be amenable to chemical synthesis, thus greatly facilitating access to a huge chemical space in the search for new commercially interesting compounds

## Background

Traditionally, discovery and production of small molecule compounds, including drugs, has been associated with organic synthetic chemistry. However, most of the existing drugs on market are derived from natural sources, either in the form of compounds purified directly from the source organisms, derivatives of such compounds, or compounds for which the basic structure was inspired by natural compounds [1,2]. Natural products are often not tractable to chemical synthesis and isolation from the natural source can be difficult, in practice limited to macroscopic organisms or species that can be grown or reared in controlled environments. Despite these problems [3], exploring microorganisms [4] and plants [5] for natural compounds is still considered among the best options for drug discovery and the potential in the area is huge.

The core technologies used to discover and develop active natural products have not changed significantly over the past decades. One way to improve the situation is by using metabolic engineering of microorganisms and some recent improvements of molecular biology techniques and combinatorial biosynthesis approaches have resulted in several examples of heterologous expression of entire prokaryotic gene clusters. The expression of functional eukaryotic pathways has been more challenging. Short eukaryotic pathways have been assembled in bacteria and yeast, combining genes from different species, mainly plants [6-9]. These studies have focused on specific pathways with one or a few specific genes for each enzymatic step cloned on plasmid vectors, and have resulted in the production of a variety of products. Although proven and straightforward, this cloning strategy has some restrictions regarding the number of genes that can be introduced and maintained at the same time in the new host, and in the limited flexibility for testing several gene combinations simultaneously.

An increasing number of reports describe the activity of flavonoids in several therapeutic areas including cancer [10,11], inflammation [12], cardiovascular disease [13], Central Nervous System disorders [14], and several others [15]. Their antiviral and antibacterial activities are also well documented [16]. This class of plant secondary metabolites displays a broad range of aromatic structures, most of which are stable, soluble and UV-active, greatly facilitating their detection. Flavonoid biosynthetic pathways have been assembled in *E. coli *[17-21] and recently production of flavonoids was achieved in yeast (*S. cerevisiae*) as well [22,23], in some cases based on precursor feeding [7,22-24].

Here we present a novel and conceptually different strategy for *in vivo *synthesis of natural compounds facilitating access to the chemistry of natural compounds, in particular from the eukaryotic world. It allows the expression of large numbers of heterologous genes, and comprises the option of combining these in a random manner. Using modified eYACs (expressible Yeast Artificial Chromosomes) for expression in yeast (*S. cerevisiae*), a host which has several advantages over bacteria for expression of eukaryotic genes [7,8,22], we were able to assemble several different, but related, biosynthetic pathways by a single step random approach. The production of diverse flavonoid compounds was used to demonstrate the potential of this technology.

## Results

### Assembly, transformation, and stable replication of eYACs

**Figure 1:**
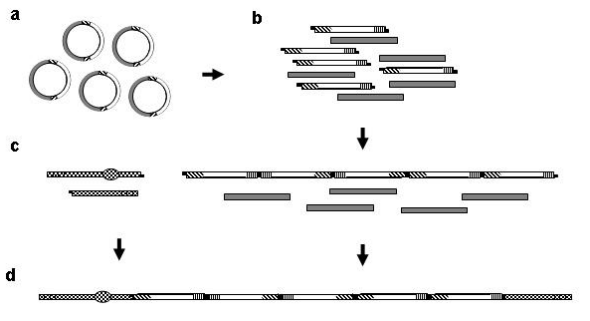
**Assembly of eYACs**. (a) Genes of interest are cloned into Entry vectors and amplified in *E. coli*. (b) Expression cassettes (hatched and white) containing the genes are generated by a double digest of Entry vectors, leaving cassettes with compatible sticky ends, while backbone DNA (gray) is left with blunt ends. (c) Cassettes are concatenated by ligation. In parallel, YAC arms (checked), supplying all necessary elements for chromosomal function, are prepared by double digest of a pYAC4 derivative. Both long and short arms have compatible, sticky overhangs at one end and telomeres at the other. The long arm also contains a centromere (checked oval) and an autonomous replicating sequence (ARS). Each arm also carries an auxotrophic selection marker (long arm *TRP1 *and short arm *LEU2*). (d) Arms are ligated to the ends of cassette concatemers to form eYACs.

**Figure 2:**
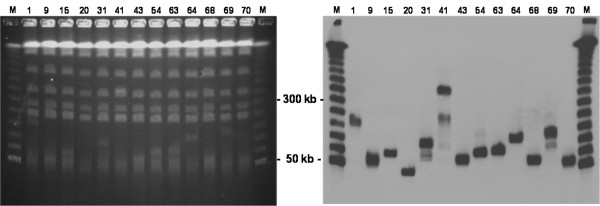
**Verification of eYACs in flavonoid producing strains**. Separation of eYACs and yeast chromosomes by PFGE (left). Numbers indicate different clones, and M is the Lambda DNA size marker (BioRad) indicating upward increments of approx. 50 kb. Settings were optimized for separation in the 50 – 600 kb area. eYACs are visible in the lower part of the gel after ethidium bromide staining. Confirmation of the eYACs by Southern DNA hybridization (right).

Gene coding sequences representing enzymes of the flavonol pathway were cloned into Entry vectors between yeast promoters and terminators (Fig. 1). To allow concerted regulation of expression, methionine dependent promoters were selected from different *Saccharomyces *species. These had previously been found to exhibit expression patterns in *S. cerevisiae *similar to the native *MET25 *(data not shown). The terminators were selected randomly among those commonly used in yeast expression vectors. After amplification in *E. coli*, the pool of Entry vectors was digested with two rare cutting restriction enzymes to release the expression cassettes from the vector backbones. Expression cassettes were then randomly concatenated, by ligation, into long chains of high molecular weight DNA. Subsequently, YAC arms containing telomeres, yeast auxotrophic markers, and yeast elements for replication and segregation were ligated onto the ends of the concatemeric DNA to create linear eYAC molecules. These eYAC molecules, each with a random combination of gene expression cassettes, were transformed into yeast (*S. cerevisiae*) by spheroplasting and selected for by means of the auxotrophic markers. The size of eYACs in transformed clones ranged from around 40 Kb to 500 Kb with an estimated average size of about 130 Kb. Assuming an average size of 2.5 Kb per expression cassette this corresponds to approximately 50 randomly combined cassettes per eYAC. In the majority of clones, eYACs were visible by simple ethidium bromide staining after gel electrophoresis, indicating relatively stable replication of these artificial chromosomes, and in all clones eYACs were easily visualized by DNA hybridization (Fig. 2). Phenotypes (flavonoid production) were retained after more than 50 generations suggesting that eYACs exhibit a level of stability comparable to normal YACs, in which the insert is typically a fragment of genomic DNA. For further confirmation of eYAC integrity, DNA was isolated from two clones and digested with *Asc*I to release the expression cassettes. These were re-cloned in *E. coli *and presence of the flavonoid genes was verified by DNA sequencing (data not shown).

### Reconstitution of flavonoid pathways

**Figure 3:**
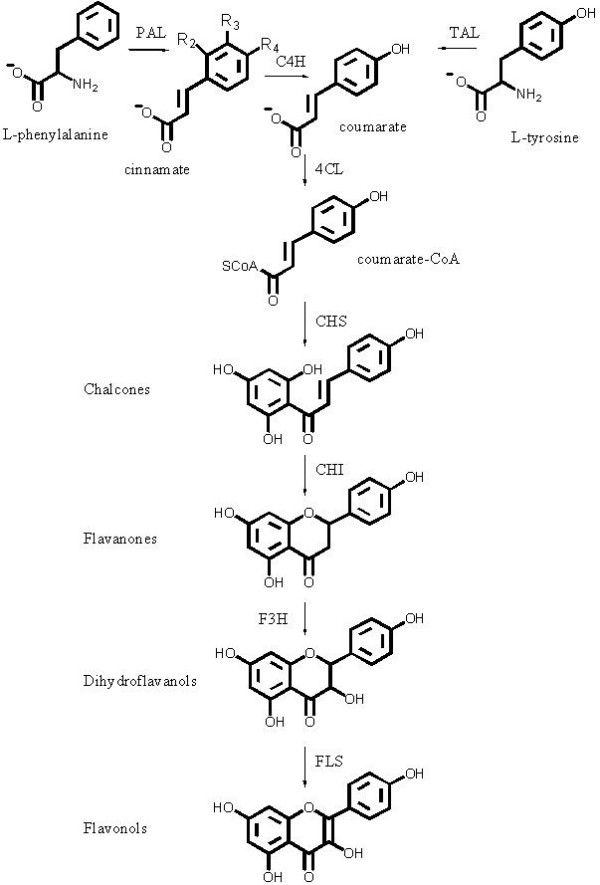
**The biosynthetic pathways reconstituted on eYACs in the FL1 library included PAL (phenylalanine amonialyase), C4H (cinnamate 4-hydroxylase), 4 CL (4 coumarate:CoA ligase), CHS (chalchone synthase), CHI (chalchone isomerase), F3H (flavanone 3-hydroxylase), and FLS (flavonol synthase)**. No specific TAL (tyrosine ammonialyase) was used, but some PAL enzymes are known to also have this function. For the FL2 library the enzymes PAL and C4H were omitted. As shown for cinnamate the phenyl ring can be differently substituted. Cinnamate has hydrogen in all 3 positions, whereas coumarate has R_4 _= OH, caffeate has R_3 _= OH; R_4 _= OH, and umbelleate has R_2 _= OH; R_4 _= OH. Some unnatural derivatives, substituted with halogens at the R_3 _and R_4 _positions, were also used as precursors in this study.

**Figure 4:**
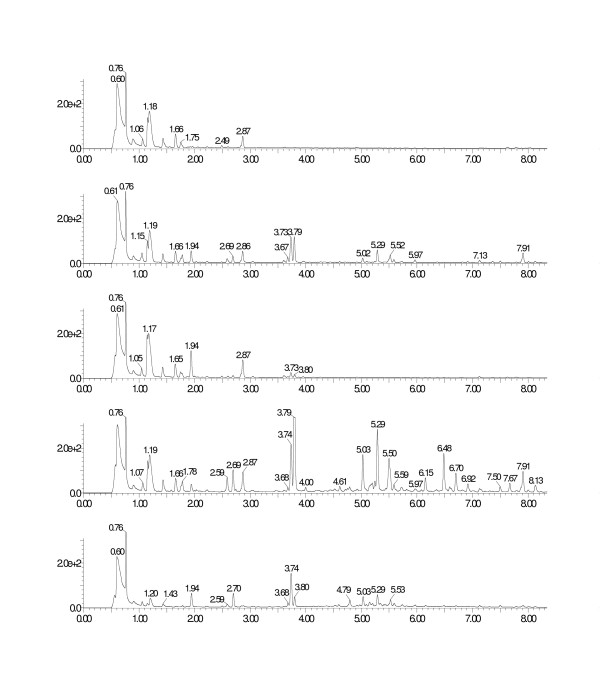
**Yeast clones from the FL1 library exhibit different expression patterns according to the combination of genes on the eYAC, as illustrated by the UV-chromatograms**. From top to bottom: control with no eYAC (only a plasmid with the selection markers *TRP1 *and *LEU2 *as for eYACs) followed by clones nos. 1, 31, 41, and 43 with eYACs of approximately 220 Kb, 130 Kb, 350 Kb, and 60 Kb, respectively (see Fig. 2). Several new UV-peaks appear after introduction of eYACs, most notably the naringenin (3.74) and kaempferol (3.79).

Genes representing the entire flavonol pathway (Fig. 3) were used to construct the FL1 library. In clones from this library various amounts of kaempferol, a flavonol, were detected in 8 out of 24 clone pellets analysed, thus confirming assembly of entire functional pathways. In addition, clones with incomplete pathways were obtained as indicated by the accumulation of intermediates such as the flavanone naringenin. More generally, we observed different expression patterns between clones (Fig. 4), indicating the assembly of pathways with either redundancies and/or differences in the gene combination on individual eYACs. Maximum total yields observed, in pellet and growth medium combined, were 858 μg/L naringenin and 235 μg/L kaempferol. Also pinocembrin (a flavanone) and dihydrokaempferol (a dihydroflavonol) were detected in some clones, as well as compounds that by UV spectra, masses, and MS-MS fragmentation data were identified as flavonoids, e.g. several with unexpected hydroxylation patterns (see Additional file 1). Such compounds are likely to be the result of the combined action of yeast metabolic and eYAC derived enzymes, as observed earlier by others [23]. Finally, expression of the flavonoid genes seems to be accompanied by specific changes in the host metabolism, leading to the appearance of several new peaks, especially in the later half of the LC chromatogram. Spectral data for all detected flavonoid compounds are available online in supplementary data (see Additional file 1).

### Diversification by precursor feeding

**Figure 5:**
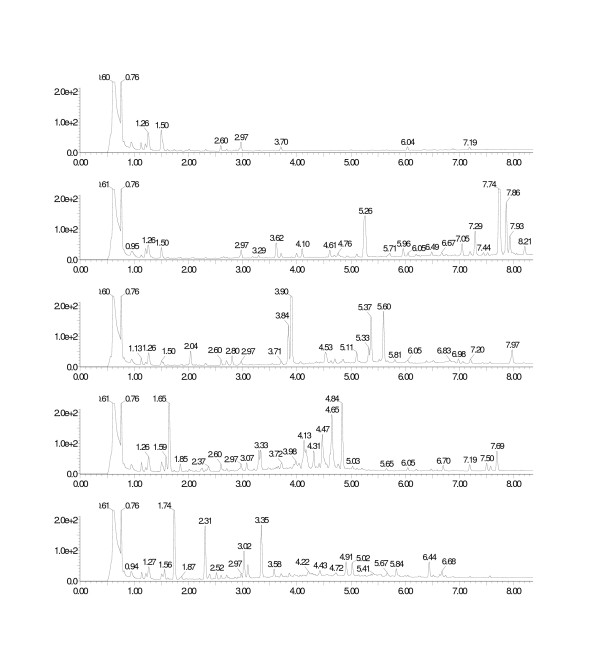
**Precursor feeding experiment with a clone from FL2 library containing a truncated flavonoid pathway (see text) on an approximately 500 Kb eYAC**. Using natural precursors, a variety of flavonoids are produced including the expected flavanone and flavanol. From top to bottom: No precursor; cinnamic acid, yielding pinocembrin (5.26) and galangin (5.26); coumaric acid, yielding naringenin (3.84) and kaempferol (3.90); caffeic acid, yielding eriodictyol (3.30) and quercitin (3.33); and finally umbellic acid, yielding 5,7,2',4'-tetrahydroxy-flavanone (3.35) and morin (3.02).

A common strategy to expand the structural diversity of products from a particular biosynthetic pathway is to use an external supply of molecular building blocks. To explore this option for expanding our repertoire of flavonoids, we prepared an additional eYAC library, FL2, in which the first steps (*PAL *and *C4H*) of the flavonol pathway were not included, preventing the use of internal yeast precursors. This would allow flavonoids to be produced only after feeding with a substrate accepted by the enzyme 4-coumarate-CoA ligase (*4CL*). Clones from the FL2 library were first grown in the presence of coumaric acid and screened by LC-UV/MS for production of the flavonoids naringenin or kaempferol. One or both of these compounds were found in about 50% of clones analysed. A clone containing an approximately 500 Kb eYAC (data not shown), and producing naringenin and kaempferol when fed with coumaric acid, was selected for precursor feeding with various natural and unnatural cinnamic acid derivatives. With no external substrate this clone did not produce any detectable flavonoids; but when fed with the natural precursors cinnamic acid, coumaric acid, caffeic acid, or umbellic acid, the flavanones and flavonols corresponding to all four precursors were produced (cinnamic acid yielded pinocembrin and galangin; coumaric acid yielded naringenin and kaempferol; caffeic acid yielded eriodictyol and quercitin; and umbellic acid yielded 5,7,2',4'-tetrahydroxy-flavanone and morin) (Fig. 5). When feeding with the halogenated precursors 4-chloro-cinnamic acid, 4-bromo-cinnamic acid, and 3-bromo-, 4-fluoro-cinnamic acid we found the corresponding halogenated flavanones in small amounts and, in addition, trace amounts of other flavonoid compounds (see Additional file 2 and 3).

## Discussion

Our results show that eYACs can be readily used for targeted reconstitution of a particular heterologous pathway in yeast. Used for this purpose the approach is simple and rapid, and the outcome to some extent similar to what has been reported by others, who used more directed cloning strategies [17,23]. However, the real potential of the eYAC approach as a metabolic engineering tool lies in the fact that many different multi-species pathways can be assembled randomly with a single procedure. In the example of the full flavonol pathways of the FL1 library, genes from at least three source organisms were required to reach the end product. It confirms the principle that genes from, in theory, any organism can be combined to achieve novel biosynthetic pathways and desired phenotypes.

The eYAC approach allow cloning and expression of large numbers of genes and, even in comparison to combinatorial biosynthesis in bacteria, the number of genes per individual cell that can be expressed in this system is extraordinarily high. With an average eYACs size of around 130 Kb, as obtained here, and an average size of 2.5 Kb per expression cassette (assuming coding sequences of ~1.5 Kb) the calculated number of heterologous genes corresponds to around 50 genes per molecule. The metabolic load of expressing such numbers of heterologous genes would potentially put the yeast under considerable stress [25]. However, with the FL1 library we observed only minor growth retardation, with an average doubling time during exponential growth increasing from 118 min during non-inducing conditions to 138 min in medium inducing expression (data not shown). As known for regular YACs, yeast is able to maintain and replicate a number of such large chromosome sized molecules, both in haploid and diploid cells. Similarly, it should therefore be possible to maintain more than one eYAC of the type described here in a haploid cell and, by mating, obtain diploids with several eYACs, together carrying hundreds of heterologous genes. We are currently exploring these options, and with e.g. 200 or more heterologous genes the issue of metabolic load may have to be investigated further. Also, spurious recombination between repeated sequences could become more frequent with increased numbers of cassettes and probably additional promoters and terminators will have to be employed to prevent this from becoming a serious issue. Several new promoters and terminators have already been cloned in our laboratory with this purpose (data not shown).

The amount of public gene sequence information from both eukaryotes and prokaryotes is increasing rapidly, and with the advent of cheap commercial gene synthesis any sequence can be optimized for expression in yeast. Homologues, and functional analogues, from different organisms will often have different substrate specificities and turnover rates. Including several different enzymes in each catalytic step of a pathway promotes natural selection of optimized combinations for a given phenotype and reduces the effect of metabolic bottlenecks. Further, inclusion of enzymes with additional modifying activities, would allow biosynthesis of a variety of intermediates and end products. Finally, the occurrence of compounds which are not direct intermediates of the introduced pathway, strongly suggests a contribution from yeast metabolic enzymes. Altogether, these features obviously increase the diversity of compounds that can be created and, along with the option of precursor feeding, give access to a broad and diverse chemistry space.

Yeast has been extensively used for construction of drug screening assays, e.g. the yeast two-hybrid model for receptor/ligand binding or protein/protein interactions [26]. Assays like these are fully compatible with the introduction of eYACs, offering the possibility for new active compounds to be produced and detected in the same cell. For any interesting compound found by such screening, chemical synthesis may often be the preferred route of production, but the current approach also offers the option of identifying and transferring relevant genes to a more "production-friendly" host strain in which yields can then be optimized.

## Conclusion

We have developed a fast and simple strategy for combining and assembling large numbers of genes deriving, in principle, from any kind of species, metabolic pathway, or functional group of enzymes. The technology is ideal for generating high numbers of structurally diverse compounds, many of which may not be amenable to chemical synthesis. By facilitating the exploration of natural products and, at the same time, providing a route to otherwise inaccessible and possibly unexpected chemistry, this technology is likely to improve the odds of drug discovery in the pharmaceutical industry.

## Methods

### Host strains

All bacterial cloning was performed in *E. coli *XL10 Gold super competent cells (Stratagene). The yeast strain used was a *S. cerevisiae *BY4742 derivative with the genotype: MATα, his3Δ1, leu2Δ0, lys2Δ0, trp1Δ, ura3Δ0.

### Construction of vectors

For ease of handling, all genes were first cloned in *E. coli *vectors having yeast expression cassettes containing *i*) a yeast promoter, *ii*) the gene of interest, and *iii*) a yeast transcription termination signal. These Entry vectors were prepared by first inserting a small synthetic multiple cloning site (MCS) between the two *Pvu*II sites of pBluescript II KS+ (Stratagene) to create pEVE1. The basic design of the MCS was *Srf*I – *Asc*I – *Bgl*II – *Hind*III – *Sfi*I(a) – *Sfi*I(b) – *Sac*II – *Sph*I – *Asc*I – *Srf*I (see Additional file 4). The vectors have a 622 bp stuffer fragment between the *Sfi*I sites. To reduce the number of repeated sequences in the final YAC expression vectors and, hence, limit the chance of spontaneous homologous recombination, a mix of Entry vectors was used, containing all possible combinations of 4 different promoters and 6 different transcription terminators, all deriving from yeast species closely related to *S. cerevisiae*. To create the mix of 24 Entry vectors, two *MET2 *and two *MET25 *promoters were cloned between *Bgl*II and *Hind*III and 6 different yeast transcription termination signals were inserted between *Sac*II and *Sph*I. A list of promoters and terminators is provided in supplementary data (see Additional file 4) online. The YAC vector used was based on pYAC4 (GenBank acc. no. U01086), in which the original *URA3 *auxotrophic marker gene had been exchanged with a *LEU2 *marker gene. Finally, an *Asc*I site had been inserted into the unique *Eco*RI site separating the two YAC arms. An outline of the vectors is provided as supplementary online data (see Additional file 5).

### Cloning in Entry vectors

Genes representing enzymes of the flavonol pathway (Fig. 3) were selected from a range of plants (parsley, soybean, maize, thale cress, bishop's weed, morning glory, petunia, kudzu, tutsan, mandarin orange, strawberry, and rice), a fungus (*Aspergillus*), and a yeast (*Rhodosporidium*). Based on published sequences the genes were either cloned from cDNA, prepared using the Mint cDNA synthesis kit from Evrogen JSC, Moscow, Ru, or they were custom synthesized by commercial suppliers (Codon Devices, MA, USA, or Epoch Biolabs, TX, USA) and, in this case, codon optimised for expression in yeast. All genes were cloned in a mix of 24 Entry vectors, between *Hind*III and *Sac*II, and 3–5 different clones were selected for each gene. Genomic DNA from salmon sperm was digested with *Hind*III and *Sph*I, and size fractionated by agarose gel electrophoresis to obtain fragments with an estimated size of 2 – 4 Kb. These fragments were cloned in *Hind*III and *Sph*I of an empty Entry vector (pEVE1) to create a library of genomic spacer fragments. The *S. cerevisiae *replication signal ARSH4 was PCR amplified from pRS413 (GenBank acc. no. U03447) and cloned in *Hind*III and *Sph*I of an empty Entry vector.

### Construction of eYACs

After amplification of Entry vectors in *E. coli *these were digested with the two rare cutting restriction enzymes *Asc*I (NEB) and *Srf*I (Stratagene), generating expression cassette fragments with sticky *Asc*I ends, and vector backbone fragments with blunt *Srf*I ends. Entry vectors containing salmon sperm DNA, and the ARSH4 sequence, were digested the same way. Reaction mixes of 500 μg total DNA were set up, for the FL1 library, containing 50 μg Entry vector of each of the 7 steps in the flavonol pathway, 5 μg ARSH4 vector, and 145 μg of vector with salmon sperm DNA and, for the FL2 library, containing 50 μg Entry vector of each of steps 3–7 of the pathway, 5 μg ARSH4 vector, and 245 μg of vector with salmon sperm DNA. The specific genes used for all steps are listed in online supplementary data (see Additional file 6 and 7). The reactions were incubated o/n with *Asc*I and *Srf*I, and the two small spacer fragments between the *Asc*I and *Srf*I sites (21/25 bases) were removed by filtration on Microcon YM-30 columns (Millipore).

Cassettes were concatenated, favoring the ligation of sticky ends, by adjusting the reaction to 5 mM ATP (Fermentas), adding T4 ligase (Stratagene) (25 mU/μg DNA starting material), and incubating for 3 h at RT, yielding high molecular weight DNA. To create fresh sticky ends for adding YAC arms, the DNA was then briefly submitted to a 3 min partial *Asc*I digest (6 mU/μg DNA starting material) before adding YAC arm DNA at a w/w ratio of 1:10, based on starting material of YAC vector and Entry vector, respectively, and performing a final ligation reaction, all in the same vial. Arm DNA had been prepared in advance by an o/n double digest of the YAC vector with *Asc*I and *Bam*HI followed by dephosphorylation and phenol/chloroform extraction.

### Size fractionation of eYACs

To select for molecules in the desired size range for transformation, newly synthesized eYACs were fractionated by Pulsed Field Gel Electrophoresis (PFGE) on a 1% low melting agarose gel (SeaPlaque, Lonza) run on a CHEF-DR III system (BioRad) set at switch angle 120°, switch time 8 sec, 6.7 Vcm^-1 ^for 16 h at 14°C. Molecules estimated to be above 50 Kb were, thus, concentrated in a compression zone which was excised and treated with β-agarase (NEB) to liberate the DNA [27,28]. Without further purification, but after estimating the DNA concentration by gel electrophoresis, the eYAC preparation was used for transformation of yeast.

### Spheroplast transformation

Transformation was done essentially as described by Green et al. [29], except that 1 L of cells were grown in YPD to an OD_600 _of 3–5 of which 3000 OD units (e.g. 1 L at OD_600 _= 3) were used for one transformation batch. Spheroplast formation was monitored by measuring the reduction of OD_600 _after dilution in water. Incubation with Zymolyase-100T (Seikagaku Corp.) was continued until the OD_600 _reached 20% of initial density. eYAC DNA in solution (see above) was added to spheroplasts at a ratio of max 20% v/v and carefully mixed. Spheroplast recovery medium was supplemented with uracil, histidine, leucine, tryptophan, adenine, lysine and methionine at 20 mg/L. Transformants were selected on SC-Leu medium, containing 1 M sorbitol. Nobel agar (Difco), at 2.5%, and CSM-Leu powder (MPBio), were used in the top agar. Transformants were restreaked as single clones in SC-Leu-Trp plates before further analysis.

### Documentation of eYACs

Yeast clones were grown in medium selective for the YAC arms and small scale preparations of agarose embedded chromosomal DNA was prepared using the LIDS procedure [28]. 40 μL agarose embedded DNA, as well as a lambda DNA size marker (BioRad), were loaded on a 1% low melting agarose gel and the DNA separated by PFGE on a CHEF-DR III system (BioRad) set at switch angle 120°C, switch time increasing from 10–40 sec., and 6.7 Vcm^-1 ^for 16 hours at 14°C. The gel was stained with ethidium bromide and photographed. The DNA was then nicked by UV-irradiation and transferred under alkaline conditions to Zeta-Probe^® ^GT nylon membrane (BioRad), according to the protocol described in the CHEF-DR III manual. Southern DNA hybridization analysis was performed using a 500 bp DNA probe specific for the *LEU2 *marker gene on the short YAC arm, together with a 400 bp probe specific for the lambda DNA size marker. Probes were generated using the PCR DIG probe synthesis kit (Roche). Hybridization was performed according to the Zeta-Probe^® ^GT Blotting Membranes Standard protocol and detection was performed using the DIG Luminescent Detection Kit (Roche).

### Flavonoid production

The promoters of expression cassettes were from either *MET2 *or *MET25 *genes to allow concerted induction of all heterologous genes by growing cells in methionine deficient medium. Yeast pre-cultures were inoculated from single colonies in SC-Leu-Trp supplemented with 2 mM methionine, for repression of heterologous gene expression, and grown for 24 h at 30°C, before being harvested by centrifugation and resuspended in SC-Leu-Trp-Met to a concentration of OD_600 _= 0.1. Cultures of 25 mL were then grown for 48 – 72 h at 30°C, centrifuged, and cell pellets were analyzed. In precursor feeding experiments cinnamic acid, or its derivatives, was added to the growing culture in five aliquots at 10–14 h intervals, reaching a final precursor concentration of 0.5 mM, minus what was consumed during the experiment.

### Extraction and LC-MS/MS analysis

Cell pellets, 150 mg, were suspended in 5 mL organic solvent (ethylacetate:dichloromethane:methanol) (3:2:1) and sonicated 1 h in an ultrasonic bath. Growth media were extracted in 1 volume of organic solvent. The organic phases were collected, dried in a SpeedVac^® ^and resuspended in 100 μL DMSO. Typically, aliquots of 5 μL were injected for LC-UV/MS and MS/MS analysis, running a 10 min gradient of mobile phases A (water:acetonitrile (95:5) + 0.1% formic acid) and B (acetonitrile + 0.1% formic acid), going from 5–100% B. Flow rate: 0.4 mL/min. Column: BEH C18 (2.1 × 100 mm) 1.7 μm, column temperature 35°C. UV detection between 210 – 400 nm. The instrument was a Waters Acquity^® ^UPLC- TQD coupled with a UV Photodiode Array Detector (PDA). LC-UV/ESI/MS were run in ionisation modes ES+ and ES-, capillary voltage 3.5 kV, cone voltage 35 V, extractor 3 V, source temperature 130°C, desolvation temperature 350°C, desolvation gas flow 800 mL/min, and cone gas flow 50 mL/min. Scanning range was m/z 90–750, and scan duration (time) 0.25 sec. LC-UV/DAD-ESI/MS/MS source parameters were the same as for the MS analysis. Collision gas was argon, collision gas flow 0.5 mL/min (1.02 × 10^-2 ^mbar), collision energy 20–40 eV, and scan time 0.1 sec. Additional analytical results are available online (see Additional file 1, 2, 3, 8, 9, 10, and 11).

## Abbreviations

YAC: Yeast Artificial Chromosome; eYAC: expressible Yeast Artificial Chromosome; PFGE: Pulsed Field Gel Electrophoresis; DMSO: Dimethyl sulfoxide; LIDS: Lithium Dodecyl Sulfate; LC-MS/MS: Liquid chromatography-tandem mass spectrometry; DAD: Diode Array Detector; ESI: Electron Spray Ionisation.

## Competing interests

The authors declare competing interests. All authors are or were employed by Evolva.

## Authors' contributions

NG, SVSN and ASS conceived the approach of using YACs. MN, CAFN, TG, TØT, ES, PK, AH, MS, OT, CF, REA, MJ and TT developed the technology and did the majority of the molecular biology and yeast engineering work. EHH identified and cloned the promoters. MM did the chemical analysis. SvSF and TB did the extractions of yeast samples. SK, PM, SS, NK, IS, and AB identified and cloned the majority of the flavonoid genes. MN planned and supervised the project. Together with TT, MM, and TØT he wrote the manuscript. All authors read and approved the final manuscript.

## Supplementary Material

Additional file 1 **Spectral data of detected compounds**. Spectral data. Click here for file

Additional file 2 **Compounds produced from halogenated precursors**. Ion chromatograms. Click here for file

Additional file 3 **Halogenated flavanones**. Ion chromatograms. Click here for file

Additional file 4 **Promoter and Terminator sequence names and accession numbers**. Overview of names and accession numbers. Click here for file

Additional file 5 **Vectors used for preparing eYACs**. Vector diagrams. Click here for file

Additional file 6 **FL1 library enzyme names and accession numbers**. Overview of names and accession numbers. Click here for file

Additional file 7 **FL2 library enzyme names and accession numbers**. Overview of names and accession numbers. Click here for file

Additional file 8 **Fragmentation patterns for identification of flavonoids**. Diagrams for fragmentation of flavonoid molecules. Click here for file

Additional file 9 **Compounds produced by FL1 pathway**. Ion chromatograms. Click here for file

Additional file 10 **Compounds produced by FL2 pathway**. Ion chromatograms. Click here for file

Additional file 11 **Supplementary references**. References. Click here for file
