# Supplementary material for: Yeast artificial chromosomes employed for random assembly of biosynthetic pathways and production of diverse compounds in Saccharomyces cerevisiae
Source: Microb Cell Fact. 2009 Aug 13;8:45. doi: 10.1186/1475-2859-8-45 (PMC2732597; doi:10.1186/1475-2859-8-45)
Supplement: Additional file 1 — Spectral data of detected compounds. Spectral data. [file 1475-2859-8-45-S1.doc]

| **Flavanones / Chalcones**  **Compound 2 (5,7,4’-trihydroxy-flavanone, naringenin)**  RT (Retention Time): **4.05 min**  UV λ_max_ nm: **286, 360sh**  LC-ESI/MS, *m/z*: **273** [M + H]^+^  LC-ESI-MS/MS, *m/z* (rel. int.): **153** ^1,3^A^+^ (100%), **179** [A – B-Ring]^+^ (3%), **147** ^1,4^B^+^ - 2H (48%), **119** ^1,4^B^+^ - 2H - CO (50%)  **Compound 2a (trihydroxy-flavanone, with presence of two hydroxyl groups in the A-Ring and presence of one hydroxyl group in the B-ring)**  RT (Retention Time): **5.44 min**  UV λ_max_ nm: **289, 360sh**  LC-ESI/MS, *m/z*: **273** [M + H]^+^  LC-ESI-MS/MS, *m/z* (rel. int.): **255** [M + H – H_2_O]^+^ (3%), **153** ^1,3^A^+^(100%), **147** ^1,4^B^+^ - 2H (42%), **119** ^1,4^B^+^ - CO (50%)  **Compound 2b (trihydroxy-flavanone, with presence of two hydroxyl groups in the A-Ring and presence of one hydroxyl group in the B-ring)**  RT (Retention Time): **5.68 min**  UV λ_max_ nm: **289, 360sh**  LC-ESI/MS, *m/z*: **273** [M + H]^+^  LC-ESI-MS/MS, *m/z* (rel. int.): **255** [M + H – H_2_O]^+^ (3%), **153** ^1,3^A^+^ (100%), **147** ^1,4^B^+^ - 2H - CO (33%), **119** ^1,4^B^+^ - CO (18%)  **Compound 4 (5,7-dihydroxy-flavanone, pinocembrin)**  RT (Retention Time): **5.43 min**  UV λ_max_ nm: **289,** **360sh**  LC-ESI/MS, *m/z*: **257** [M + H]^+^  LC-ESI-MS/MS, *m/z* (rel. int.): **153** ^1,3^A^+^ (100%), **131** ^1,4^B^+^ - 2H (20%), **103** ^1,4^B^+^ - 2H – CO (48%)  **Compound 4a (dihydroxy-flavanone, with presence of two hydroxyl groups in the A-Ring)**  RT (Retention Time): **7.18 min**  UV λ_max_ nm: **294**  LC-ESI/MS, *m/z*: **257** [M + H]^+^  LC-ESI-MS/MS, *m/z* (rel. int.): **153** ^1,3^A^+^ (100%), **131** ^1,4^B^+^ - 2H (33%), **103** ^1,4^B^+^ - 2 H - CO (55%)  **Compound 4b (dihydroxy-flavanone, with presence of two hydroxyl groups in the A-Ring)**  RT (Retention Time): **7.7 min**  UV λ_max_ nm: **295**  LC-ESI/MS, *m/z*: **257** [M + H]^+^  LC-ESI-MS/MS, *m/z* (rel. int.): **153** ^1,3^A^+^ (100%), **131** ^1,4^B^+^ - 2H (23%), **103** ^1,4^B^+^ - CO (58%)  **Compound 6 (5,7,3’,4’-tetrahydroxy-flavanone, eriodyctiol)**  RT (Retention Time): **3.3 min**  UV λ_max_ nm: **286, 360sh**  LC-ESI/MS, *m/z*: **289** [M + H]^+^  LC-ESI-MS/MS, *m/z* (rel. int.): **179** [A – B-Ring]^+^ (5%), **163** ^1,4^B^+^ - 2H (35%), **153** ^1,3^A^+^ (100%), **135** ^1,4^B^+^ - 2H - CO (10%)  **Compound 6a (pentahydroxy-chalcone, with presence of three hydroxyl groups in the A-Ring and two hydroxyl groups in the B-ring)**  RT (Retention Time): **4.57 min**  UV λ_max_ nm: **250sh, 300sh, 374**  LC-ESI/MS, *m/z*: **289** [M + H]^+^  LC-ESI-MS/MS, *m/z* (rel. int.): **179** [A – B-Ring]^+^ (6%), **163** ^1,4^B^+^ - 2H (55%), **153** ^1,3^A^+^ (100%), **135** ^1,4^B^+^ - 2H - CO (16%) |
| --- |

**Additional file 1**. Spectral data of detected compounds.

| **Flavanones / Chalcones (continued)**  **Compound 6b (pentahydroxy-chalcone, with presence of three hydroxyl groups in the A-Ring and two hydroxyl groups in the B-ring)**  RT (Retention Time): **4.69 min**  UV λ_max_ nm: **245sh, 387**  LC-ESI/MS, *m/z*: **289** [M + H]^+^  LC-ESI-MS/MS, *m/z* (rel. int.): **179** [A – B-Ring]^+^ (6%), **163** ^1,4^B^+^ - 2H (56%), **153** ^1,3^A^+^ (100%), **135** ^1,4^B^+^ - 2H – CO (15%)  **Compound 6c (pentahydroxy-chalcone, with presence of three hydroxyl groups in the A-Ring and two hydroxyl groups in the B-ring)**  RT (Retention Time): **4.99 min**  UV λ_max_ nm: **245sh, 387**  LC-ESI/MS, *m/z*: **289** [M + H]^+^  LC-ESI-MS/MS, *m/z* (rel. int.): **163** ^1,4^B^+^ - 2H (25%), **153** ^1,3^A^+^ (100%), **135** ^1,4^B^+^ - 2H - CO (14%)  **Compound 8 (2’,4’,6’,2,4-pentahydroxy-chalcone)**  RT (Retention Time): **3.28 min**  UV λ_max_ nm: **260sh, 385**  LC-ESI/MS, *m/z*: **289** [M + H]^+^  LC-ESI-MS/MS, *m/z* (rel. int.): **289** [M + H]^+^ (48%), **271** [M + H – H_2_O]^+^ (38%), **163** ^1,4^B^+^ - 2H (30%), **153** ^1,3^A^+^ (100%), **135** ^1,4^B^+^ - 2H - CO (5%)  **Compound 10 (5,7,2’,4’-tetrahydroxy-flavanone)**  RT (Retention Time): **3.4 min**  UV λ_max_ nm: **287, 330sh**  LC-ESI/MS, *m/z*: **289** [M + H]^+^  LC-ESI-MS/MS, *m/z* (rel. int.): **271** [M + H – H_2_O]^+^ (8%), **163** ^1,4^B^+^ - 2H (12%), **153** ^1,3^A^+^ (100%), **135** ^1,4^B^+^ - 2H - CO (11%)  **Compound 13 (5,7-dihydroxy-4’-chloroflavanone)**  RT (Retention Time): **6.07min**  UV λ_max_ nm: **287,350sh**  LC-ESI/MS, *m/z*: **291** [M + H]^+^  LC-ESI-MS/MS, *m/z* (rel. int.): **165** ^1,4^B^+^ - 2H (30%), **153** ^1,3^A^+^ (100%), **137** ^1,4^B^+^ - 2H - CO (12%)  **Compound 14 (5,7-dihydroxy-3’-bromo-4’-fluoro flavanone)**  RT (Retention Time): **6.2 min**  UV λ_max_ nm: **289, 335sh**  LC-ESI/MS, *m/z*: **353** [M + H]^+^  LC-ESI-MS/MS, *m/z* (rel. int.): **335** [M + H – H_2_O]^+^ (3%), **227** ^1,4^B^+^ - 2H (32%), **199** [^1,4^B^+^ - 2H - CO (21%), **153** ^1,3^A^+^ (100%)  **Compound 15 (5,7-dihydroxy-4’bromoflavanone)**  RT (Retention Time): **6.5 min**  UV λ_max_ nm: **293, 335sh**  LC-ESI/MS, *m/z*: **335** [M + H]^+^  LC-ESI-MS/MS, *m/z* (rel. int.): **209** ^1,4^B^+^ - 2H (18%), **199** ^1,3^B^+^ (100%), **153** ^1,3^A^+^ (30%) |
| --- |

**Additional file 1**. Spectral data of detected compounds.

| **Dihydroflavonols**  **Compound 1 (5,7,4’-trihydroxy-dihydroflavonol, dihydrokaempferol)**  RT (Retention Time): **3.01 min**  UV λ_max_ nm: **290, 330sh**  LC-ESI/MS, *m/z*: **289**[M + H]^+^  LC-ESI-MS/MS, *m/z* (rel. int.): **215** [M + H – H_2_O – 2CO]^+^ (25%), **197** [M+H – 2H_2_O – 2CO]^+^ (12%), **153** ^1,3^A^+^ (73%), **149** ^0,2^A^+^ - H_2_O (90%), **163** ^1,4^B^+^ (2%), **107** tropylium Ion (100%)  **Compound 1a trihydroxy-dihydroflavonol (with presence of two hydroxyl groups in the A-Ring and presence of one hydroxyl in the B-ring)**  RT (Retention Time): **4.7 min**  UV λ_max_ nm: **294, 350sh**  LC-ESI/MS, *m/z*: **289**[M + H]^+^  LC-ESI-MS/MS, *m/z* (rel. int.): **215** [M + H – H_2_O – 2CO]^+^ (20%), **197** [M + H – 2H_2_O – 2CO]^+^ (10%), **153** ^1,3^A^+^ (100%), **149** ^0,2^ A^+^ - H_2_O (50%), **107** tropylium Ion (45%)  **Compound 1b trihydroxy-dihydroflavonol (with presence of two hydroxyl groups in the A-Ring and presence of one hydroxyl in the B-ring)**  RT (Retention Time): **5.03 min**  UV λ_max_ nm: **296, 350sh**  LC-ESI/MS, *m/z*: **289**[M + H]^+^  LC-ESI-MS/MS, *m/z* (rel. int.): **215** [M + H – H_2_O – 2CO]^+^ (40%), **197** [M + H – 2H_2_O – 2CO]^+^ (15%), **153** ^1,3^A^+^ (100%), **167** ^0,2^A^+^ (10%), **149** ^0,2^A^+^ - H_2_O (70%), **107** tropylium Ion (78%)  **Compound 11 (5,7-dihydroxy-dihydroflavonol, dihydrogalangin)**  RT (Retention Time): **4.22 min**  UV λ_max_ nm: **286, 360sh**  LC-ESI/MS, *m/z*: **273** [M + H]^+^  LC-ESI-MS/MS, *m/z* (rel. int.): **199** [M + H – H_2_O – 2CO]^+^ (70%), **181** [M + H – 2H_2_O – 2CO]^+^ (12%), **153** ^1,3^A^+^ (95%), **149** [^0,2^ A- H_2_O]^+^ (100%), **147** ^1,4^B^+^ (68%), **91** tropylium Ion (51%)  **Compound 12 (5,7,3’,4’-tetrahydroxy-dihydroflavonol, taxifolin)**  RT (Retention Time): **2.55 min**  UV λ_max_ nm: **296, 350sh**  LC-ESI/MS, *m/z*: **305** [M + H]^+^  LC-ESI-MS/MS, *m/z* (rel. int.): **231** [M + H – H_2_O – 2CO]^+^ (39%), **213** [M+H – 2H_2_O – 2CO]^+^ (15%), **153** ^1,3^A^+^ (100%), **167** ^0,2^ A^+^ (50%), **161** ^1,4^ B^+^ - H_2_O (70%) |
| --- |

**Additional file 1. Spectral data of detected compounds.**
