# Supplementary material for: Yeast artificial chromosomes employed for random assembly of biosynthetic pathways and production of diverse compounds in Saccharomyces cerevisiae
Source: Microb Cell Fact. 2009 Aug 13;8:45. doi: 10.1186/1475-2859-8-45 (PMC2732597; doi:10.1186/1475-2859-8-45)
Supplement: Additional file 2 — Compounds produced from halogenated precursors. Ion chromatograms. [file 1475-2859-8-45-S2.doc]

**Additional file 2. Compounds produced from halogenated precursors.** Selected ion chromatograms of expected intermediates, after precursor feeding with halogenated cinnamic acids. The MS spectral data showed typical isotopic abundance of halogenated compounds. Compounds indicated are **13**: 5,7-dihydroxy -4’-chloroflavanone, **14**: 5,7-dihydroxy-3’-bromo-4’fluoroflavanone, and **15**: 5,7-dihydroxy -4’-bromoflavanone.
