# Supplementary material for: Yeast artificial chromosomes employed for random assembly of biosynthetic pathways and production of diverse compounds in Saccharomyces cerevisiae
Source: Microb Cell Fact. 2009 Aug 13;8:45. doi: 10.1186/1475-2859-8-45 (PMC2732597; doi:10.1186/1475-2859-8-45)
Supplement: Additional file 3 — Halogenated flavanones. Ion chromatograms. [file 1475-2859-8-45-S3.doc]

**Additional file 3. LC-ESI-MS/MS spectra of the different halogenated flavanones.** Compounds are **13:** 5,7-dihydroxy -4’-chloroflavanone; **14**: 5,7-dihydroxy-3’-bromo-4’fluoroflavanone; **15:** 5,7-dihydroxy -4’-bromo-flavanone.
