# Supplementary material for: Yeast artificial chromosomes employed for random assembly of biosynthetic pathways and production of diverse compounds in Saccharomyces cerevisiae
Source: Microb Cell Fact. 2009 Aug 13;8:45. doi: 10.1186/1475-2859-8-45 (PMC2732597; doi:10.1186/1475-2859-8-45)
Supplement: Additional file 4 — Promoter and Terminator sequence names and accession numbers. Overview of names and accession numbers. [file 1475-2859-8-45-S4.doc]

**Additional file 4.** Promoter and Transcription Terminator sequences used in the Entry vectors. Promoters and terminators were amplified by PCR on genomic DNA and cloned into *Bgl*II / *Hind*III and *Sac*II / *Sph*I, respectively of the MCS (multiple cloning site), and genes were cloned in *Hind*III / *Sac*II. The sequence of the MCS (omitting the 622 bp stuffer fragment) is: 5’-gcccgggcagttcaggctcatc

aggcgcgccatgcagggatatcagatcttggccacaattgctcgagaagcttggcctgcagggcc.....(stuffer)..…ggccgttcaggcctc

gaggccgttcaggctcgacccggggatccgcggccgcaggcctaaattgatctagagcatgcccatgggttaactgatcaatgcatcctgcatgg

cgcgcctgatgagcctgaactgcccgggc-3’.
