# Supplementary material for: Yeast artificial chromosomes employed for random assembly of biosynthetic pathways and production of diverse compounds in Saccharomyces cerevisiae
Source: Microb Cell Fact. 2009 Aug 13;8:45. doi: 10.1186/1475-2859-8-45 (PMC2732597; doi:10.1186/1475-2859-8-45)
Supplement: Additional file 5 — Vectors used for preparing eYACs. Vector diagrams. [file 1475-2859-8-45-S5.doc]

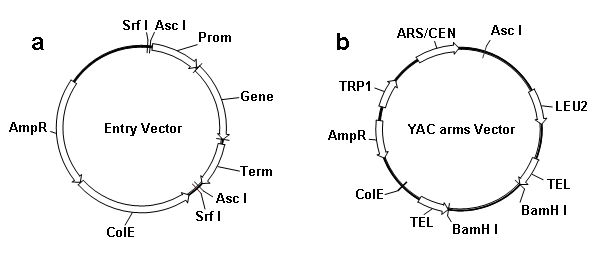


**Additional file 5. Vectors used for preparing eYACs.** (**a**) The Entry vector for cloning the flavonoid genes. Prom can be any promoter that give expression in yeast, and Term any transcription termination signal which is functional in yeast. A digestion of the vector with *Srf*I and *Asc*I will generate expression cassettes with sticky ends, and backbone fragments with blunt ends. (**b**) The YAC vector for preparing the arms is based on pYAC4. Both arms have a selectable marker and a telomere at the 3’-end. In addition, the long arm has the ARS/CEN signal for replication and segregation in yeast, plus the ColE and AmpR for amplification and selection in *E. coli*. The *URA3* gene of the original pYAC4 has been replaced by a *LEU2* gene in the short arm. An *Asc*I site was inserted into the original *Eco*RI site to make arms compatible with concatemers of expression cassettes from the Entry vector, and the arms can be released by an *Asc*I and *Bam*HI double digest.
