# Supplementary material for: Yeast artificial chromosomes employed for random assembly of biosynthetic pathways and production of diverse compounds in Saccharomyces cerevisiae
Source: Microb Cell Fact. 2009 Aug 13;8:45. doi: 10.1186/1475-2859-8-45 (PMC2732597; doi:10.1186/1475-2859-8-45)
Supplement: Additional file 6 — FL1 library enzyme names and accession numbers. Overview of names and accession numbers. [file 1475-2859-8-45-S6.doc]

**Additional file 6.** List of enzymes used to prepare eYACs for the FL1 library, allowing the reconstitution of full length flavonol pathways. CPR1 was included as a co-factor for C4H. *Two *A. thaliana* genes and the CPR1 gene were cloned from in-house cDNA libraries. The remaining genes were synthesized by commercial vendors Codon Devices, MA, USA. (codon dev.) or Epoch Biolabs, TX, USA (epoch bio.) based on DNA sequences corresponding to the listed protein IDs. These sequences were optimized by the manufacturer for expression in yeast (*S. cerevisiae*).
