# Supplementary material for: Yeast artificial chromosomes employed for random assembly of biosynthetic pathways and production of diverse compounds in Saccharomyces cerevisiae
Source: Microb Cell Fact. 2009 Aug 13;8:45. doi: 10.1186/1475-2859-8-45 (PMC2732597; doi:10.1186/1475-2859-8-45)
Supplement: Additional file 7 — FL2 library enzyme names and accession numbers. Overview of names and accession numbers. [file 1475-2859-8-45-S7.doc]

**Additional file 7.** List of enzymes used to prepare the eYACs of the FL2 library, allowing the reconstitution of truncated flavonol pathways. CPR1 was included as a co-factor for C4H. Origin of genes as described in Additional file 6.
