# Supplementary material for: Yeast artificial chromosomes employed for random assembly of biosynthetic pathways and production of diverse compounds in Saccharomyces cerevisiae
Source: Microb Cell Fact. 2009 Aug 13;8:45. doi: 10.1186/1475-2859-8-45 (PMC2732597; doi:10.1186/1475-2859-8-45)
Supplement: Additional file 8 — Fragmentation patterns for identification of flavonoids. Diagrams for fragmentation of flavonoid molecules. [file 1475-2859-8-45-S8.doc]

**Additional file 8. Fragmentation patterns used for identification of flavonoids.** The UV/DAD spectra of flavanoids showed characteristic UV absorption and allowed the categorisation of the flavonoids into subgroups. Following this peak identification process, a further LC/MS/MS analysis allowed the confirmation of the different flavanoids present in the mixture. The cleavage of the C-C bond of the C-ring, results in diagnostic ions ^1,3^A^+^ and ^1,3^B^+^, which provide information on the number and type of substituent in the A- and B-rings. According to the nomenclature of Ma, A and B represent the intact ring while the superscript on the left indicate the broken bonds of the protonated molecule. The fragment ions provide information about the subgroup to which the flavanoid belongs, as the various classes of flavanoids have specific fragmentation pattern by which they can be differentiated, and loss of small groups from the [M + H]+ ion such as H_2_O, CO, C_2_H_2_O, can also be helpful for the identification of the C-ring structure (See additional file 11 for references). The standards used for studying the fragmentation pattern were the following: chalcone: 2’,4’,6’,3,4-pentahydroxychalcone (Extrasynthèse); Flavanones: pinocembrin (Extrasynthèse), (+/-)-naringenin (Sigma), eriodyctiol (Fluka); Dihydroflavonols: (+/-)-taxifolin (sigma), Flavonols: galangin (Aldrich), kaempferol (Fluka), quercetin hydrate 95% (Aldrich), and Morin hydrate (Fluka). Each standard was diluted in MeOH, at a concentration of 0.1 mg/mL.
