# Supplementary material for: Yeast artificial chromosomes employed for random assembly of biosynthetic pathways and production of diverse compounds in Saccharomyces cerevisiae
Source: Microb Cell Fact. 2009 Aug 13;8:45. doi: 10.1186/1475-2859-8-45 (PMC2732597; doi:10.1186/1475-2859-8-45)
Supplement: Additional file 9 — Compounds produced by FL1 pathway. Ion chromatograms. [file 1475-2859-8-45-S9.doc]

**Additional file 9. Compounds produced by the full length pathway.** Selected ion chromatograms for intermediates and end products detected in the full length flavonol pathway of the FL1 library. Besides the expected molecules, some strains synthesized several different tri-hydroxylated flavanones, dihydroflavonols, and flavonols. A total of 31 compounds were characterized. 9 of them were unambiguously identified by comparing retention times, UV and MS/MS spectra with authentic standards, while the other structures were characterized on the basis of their UV spectra and fragmentation patterns. In fact, the LC-MS/MS spectra obtained from the fragmentation of the different flavanoids provided specific information on the substitution patterns of the A- and B- rings (see Additional file 1). Compounds indicated above are **1**: dihydrokaempferol (RT = 3.01 min), **1a:** tri-hydroxy-dihydroflavonol (RT = 4.7 min), **1b**: tri-hydroxy-dihydroflavonol (RT = 5.03 min), **2**: naringenin (RT = 4.05 min), **2a**: tri-hydroxy-flavanone (RT = 5.44 min), **2b**: tri-hydroxy-flavanone (RT = 5.68 min), **3**: kaempferol (RT = 4.12 min), **3a**: tri-hydroxy-flavonol (RT = 5.5 min), **3b**: tri-hydroxy-flavonol (RT = 6.32), **4**: pinocembrin (RT = 5.43 min), **5**: galangin (RT = 5.45 min), and **11**: dihydro-galangin (RT = 4.22 min).
