# Supplementary material for: Yeast artificial chromosomes employed for random assembly of biosynthetic pathways and production of diverse compounds in Saccharomyces cerevisiae
Source: Microb Cell Fact. 2009 Aug 13;8:45. doi: 10.1186/1475-2859-8-45 (PMC2732597; doi:10.1186/1475-2859-8-45)
Supplement: Additional file 10 — Compounds produced by FL2 pathway. Ion chromatograms. [file 1475-2859-8-45-S10.doc]

**Additional file 10. Compounds produced from natural precursors.** Selected ion chromatograms for the expected intermediates and end products of the FL2 library pathways. The yeast, after being supplied with different hydroxycinnamic acid derivatives, synthesized the corresponding flavonoid compounds. As for the full length pathway these strains produced several unexpected dihydroflavonols, flavanones and flavonols (see Additional file 1). Compounds indicated above are **1**: dihydrokaempferol (RT = 3.01 min), **1a:** tri-hydroxy-dihydroflavonol (RT = 4.7 min), **1b**: tri-hydroxy-dihydroflavonol (RT = 5.03 min), **2**: naringenin (RT = 4.05 min), **2a**: tri-hydroxy-flavanone (RT = 5.44 min), **2b**: tri-hydroxy-flavanone (RT = 5.68 min), **3**: kaempferol (RT = 4.12 min), **3a**: tri-hydroxy-flavonol (RT = 5.5 min), **4**: pinocembrin (RT = 5.43 min), **4a**: di-hydroxy-flavanone (RT = 7.18 min), **4b**: di-hydroxy-flavanone (RT = 7.7 min), **5**: galangin (RT = 5.45 min), **6**: Eriodyctiol (RT = 3.3 min), **6a**: penta-hydroxy-chalcone (RT = 4.57 min), **6b**: penta-hydroxy-chalcone (RT = 4.69 min), **6c**: penta-hydroxy-chalcone (RT = 4.99 min), **7**: quercetin (3.51 min), **7a**: tetra-hydroxy-flavonol (RT = 4.7 min), **8:** 2’,4’,6’,2,4,-pentahydroxychalcone (RT = 3.28), **9**: morin (RT = 3.11 min), **10:** 5,7,2’,4’-pentahydroxy-flavanone (3.4 min), **11**: dihydrogalangin (RT = 4.22 min), and **12**: taxifolin (RT = 2.55 min).
