# Supplementary material for: Yeast artificial chromosomes employed for random assembly of biosynthetic pathways and production of diverse compounds in Saccharomyces cerevisiae
Source: Microb Cell Fact. 2009 Aug 13;8:45. doi: 10.1186/1475-2859-8-45 (PMC2732597; doi:10.1186/1475-2859-8-45)
Supplement: Additional file 11 — Supplementary references. References. [file 1475-2859-8-45-S11.doc]

**Additional file 11. Supplementary references**

1. Markham KR: *Techniques of flavanoid identification.* London: Academic Press; 1982

2. Cuyckens F & Claeys M: **Mass spectrometry in the structural analysis of flavanoids.** *Journal of Mass Spectrometry* 2004, **39**:1-15

3. Ma YL, Li QM, Van den Heuvel H, Claeys M: **Characterization of flavone and flavonol aglycones by collision-induced dissociation tandem mass spectrometry.** *Rapid Communications in Mass Spectrometry* 1997, **11**:1357-1364

4. Hedin PA & Phillips VA: **Electron Impact mass spectral analysis of flavanoids.** *Journal of Agricultural and Food Chemistry* 1992, **40**:607-611.

5. Tsimogiannis D, Samiotaki M, Panayotou G, Oreopoulou V: **Characterization of Flavanoids subgroups and hydroxyl substitution by HPLC-MS/MS.** *Molecules* 2007, **12**:593-606.
